# Current potential therapeutics of amyotrophic lateral sclerosis

**DOI:** 10.3389/fneur.2024.1402962

**Published:** 2024-04-24

**Authors:** Lijun Lu, Youqing Deng, Renshi Xu

**Affiliations:** ^1^Department of Neurology, The First Hospital of Nanchang, Nanchang, Jiangxi, China; ^2^Department of Neurology, Jiangxi Provincial People’s Hospital, The First Affiliated Hospital of Nanchang Medical College, National Regional Medical Center for Nervous Disease, Xiangya Hospital of Central South University, Jiangxi Hospital, Nanchang, Jiangxi, China

**Keywords:** amyotrophic lateral sclerosis, gene therapy, neuroprotective therapy, stem cell, combination therapy

## Abstract

Amyotrophic lateral sclerosis (ALS) is a debilitating motor neurological disorder for which there is still no cure. The disease seriously jeopardizes the health and lifespan of adult populations. The authors extensively retrieved the current literature about clinical and experimental ALS treatments. Based on them, this review primarily focused on summarizing the current potential clinical usage and trialing therapeutics of ALS. Currently, the clinical ALS treatments have focused primarily on relieving symptoms to improve the quality of life yet. There are a number of therapeutic approaches such as medicine, gene therapy, neuron protectants, combination therapy and stem cells. Among them, Stem cells including embryonic stem cells, mesenchymal stem cells, neural stem cells, and many other types of stem cells have been used in ALS treatment, and although the short-term efficacy is good, it is worth exploring whether this improved efficacy leads to prolonged patient survival. In addition, the supportive treatments also exert an important effect on improving the quality of life and prolong the survival of ALS patients in absence of effectively care for stopping or reversing the progression of ALS.

## 1 Introduction

Amyotrophic lateral sclerosis (ALS) is a neurodegenerative disease that causes the loss of upper and lower motor neurons, ALS patients mainly are with muscle weakness, stiffness and atrophy. Although the disease usually does not affect the patient’s intellect or senses at early stage, it may eventually lead to generalized paralysis, the impairment of multiple nervous systems and the loss of respiratory function. As a chronic progressive disease, ALS has progressively worsening symptoms that usually lead to severe disability within 3–5 years. Although some people can survive longer than the period of disease course, most patients eventually die from the respiratory dysfunction. ALS has an incidence of about 2.5 per 100,000 people and has a poor prognosis with an average survival of 3–5 years. Although ALS primarily affects motor neurons, many patients experience other symptoms such as difficulty swallowing, speech disorders, and breathing difficulties. In addition, some patients may experience some mood swings or mild cognitive impairment (1).

While the etiology of most ALS patients is unknown, about 10% of patients are linked to genetic factors. In addition, a number of environmental factors, such as long-term exposure to certain chemicals or heavy metals, have been suggested as possible links to the development of the disease (2). Based on genetics, ALS can be categorized into familial and sporadic types, with the former accounting for about 10% of cases and the latter for about 90%. More than 30 ALS-related genes have been identified, among them, the more common genes are superoxide dismutase 1 (*SOD1*), chromosome 9 open reading frame 72 (*C9orf72*), fused in sarcoma (*FUS*), and TAR DNA-binding protein (*TDP-43/TARDBP*), which are involved in a variety of cellular functions such as RNA metabolism, protein folding, autophagy and inflammation (3).

Although we have gained some understanding of some genetics and molecular mechanisms of ALS, there are still many unknowns in this area. More in-depth studies are needed to identify potential therapeutic targets and provide guidance for future drug development. Given the complexity of ALS, multiple strategies may be needed to effectively combat the disease. This includes not only medications, but also gene therapy, stem cell therapy, neuroprotective strategies, supportive and symptomatic treatment. This further emphasizes the urgency of in-depth research to develop a comprehensive treatment approach.

## 2 Available medications

### 2.1 Single medications of medicine

Riluzole is the drug that prolongs survival in ALS patients, with the greatest benefit from early use (4), but does not improve motor function, muscle strength or respiratory function. Results from two pivotal experimental studies of Riluzole show that Riluzole slightly delays the onset of respiratory dysfunction and prolongs patient survival by approximately 2–3 months (5, 6). In subsequent studies, several real-world population studies comparing Riluzole with ALS patients not using Riluzole have found significant differences in the median survival between two groups, ranging from 6 to 19 months (7). In addition, Riluzole may be the most effective at the advanced respiratory stage of ALS (8–10). The mechanism of Riluzole effects in the treatment of ALS remains to be elucidated and may be related to the inhibition of glutamate release, the reduction of neuronal toxicity response, the stabilization of neuronal membranes and the blockade of voltage-sensitive sodium channels (11) to reduce the neuronal excitability. Riluzole might be able to exert the transient effects on the cortical and axonal hyperexcitability, potentially accounting for the modest clinical effectiveness in ALS. Some ALS patients may experience the elevated liver function enzymes with Riluzole, which are more common in the high-dose patients and are reversible after discontinuation of the drug, and some patients may experience the gastrointestinal symptoms, mild malaise or weakness (6), the decreased lung function, neutropenia and other adverse effects. Therefore, while using Riluzole, we need to pay attention to monitoring liver function enzymes, gastrointestinal tract, lung function and blood cells.

Free radical-induced oxidative stress involving not only motor neuron degeneration but also glial and endothelial cell dysfunction may be an important factor in the progression of ALS, and may be further exacerbated by nutritional deficiencies, cachexia, and psychological stress as ALS progresses (12, 13). Edaravone was the first drug shown to inhibit motor deterioration in ALS since Charcot firstly described ALS nearly 150 years ago. Edaravone is a free radical scavenger that may protect neurons from oxidative stress damage by scavenging free radicals and inhibiting neuroinflammatory responses (14–16). Edaravone slows the functional decline by about 33% in ALS patients (17), originally developed as intravenous (IV) therapy for acute ischemic stroke. Based on an open-label phase 2 study, the use of Edaravone resulted in a significant reduction in the change of Revised ALS Functional Rating Scale (ALSFRS-R) scores in the patients with ALS. Moreover, Edaravone reduced 3-nitrotyrosine concentrations in the cerebrospinal fluid of ALS patients (16, 18). Some investigators have conducted a placebo-controlled phase 3 studies of Edaravone for more than 24 weeks, however, the results showed that Edaravone did not show a significant difference in ALSFRS-R scores compared to placebo, while the *post hoc* analyses of these data showed that greater effects were demonstrated in the ALS patients who met the specific following enrollment criteria: 2 scores or more on all items of ALSFRS-R, at least 80% of forced vital capacity at baseline, definite or probable ALS diagnosed by El Escorial and revised Airlie House criteria, and the disease duration of 2 years or less (19).

To validate the safety and efficacy of Edaravone effects of this *post hoc* analysis, an investigator conducted a randomized, double-blind, placebo-controlled, phase III trial in Japan, which recruited ALS patients aged 20–75 years old who met the enrollment criteria between November 28, 2011 and September 3, 2014, at 31 hospitals. The results show that Edaravone demonstrated efficacy in a small group of ALS patients who met the screening criteria for the *post hoc* analysis, with a significantly smaller decrease in ALSFRS-R scores compared to placebo (20). Edaravone was eventually initially approved for ALS treatment in Japan in 2015, and was approved by the U.S. Food and Drug Administration (FDA) in May 2017 for the treatment of ALS. It is important to note that the effects appear to be more pronounced in earlier, younger patients and those with slower disease progression. The possible side effects of Edaravone include allergic reactions, rash, itching, breathing difficulty, the swelling of face, bruises or contusions, gait disturbance, headache, dermatitis, the decreased numbers of platelets, red blood cells and white blood cells, and the elevated creatine kinase levels.

In the CENTAUR trial which was a placebo-controlled, double-blind, multicenter and randomized study, the effectiveness and safety of sodium phenylbutyrate-taurursodiol (PB-TURSO) were confirmed in the patients with ALS (21). Despite the absence of phase 3 clinical trial data, PB-TURSO has received a conditional approval for treatment in Canada in June 2022, the trade name is Albrioza. PB-TURSO was fully approved in the United States in September 2022, the trade name is Relyvrio. PB-TURSO was administered in a sachet containing fixed-dose co-formulation of 3 g of PB and 1 g of TURSO (AMX0035). PB is utilized for lowering ammonia levels in certain types of urea cycle disorders and short-chain fatty acid disorders. As a pro-drug, it is quickly converted into phenylacetate, which then acetylates glutamine, resulting in the formation of phenylacetylglutamine. In cellular models, PB may affect the ALS disease progression by altering transcription, reducing neuroinflammation and improving cellular energy metabolism (22). In animal experiments, PB alleviated histone hypoacetylation, leading to a delay in the development of motor deficits, an extension of survival time, and the enhancements of motor function (23, 24). In the United States, TURSO is referred as taurursodiol, while in Canada and Europe, it goes by the name ursodoxicoltaurine. Additionally, TURSO is frequently used as an alternative term for TUDCA, which stands for tauroursodeoxycholic acid, a derivative of ursodeoxycholic acid (UDCA) that includes taurine. TURSO, a hydrophilic secondary bile acid, is formed when taurine is conjugated to ursodeoxycholic acid. While it is primarily produced in liver, the synthesis of TUDCA can also occur within brain (25). TURSO exhibits effects that prevent cell death and reduce inflammation by inhibiting the permeability of mitochondrial membrane (26). In a cellular model for *SOD1* neurodegeneration, TURSO conjugated with glycine diminishes oxidative stress and neuroinflammation through the reduction of nitrite production and the inhibition of matrix metallopeptidase 9 activation (27). Nevertheless, the rationale for PB-TURSO in the treatment of ALS remains to be clarified, as the transcriptional and metabolic effects of the combination differ significantly from those of PB or Turso alone. It is possible that the combined action of PB-TURSO may involve processes such as nucleic acid metabolism, RNA processing, nucleoplasmic transfer, unfolded protein response, mitochondrial function, and innate immune function, which need to be confirmed by further studies. However, the recent failure of the AMX0035 and TUDCA trials was reported. AMX0035 received US FDA approval in September 2022. However, oral treatment with AMX0035 and TUDCA failed to meet primary clinical and electrodiagnostic endpoints in clinical trials. Despite this failure, a number of exploratory endpoints included in phase 2/3 trials suggest AMX0035 and TUDCA has the potential to significantly slow clinical worsening, improve quality of life, and prolong survival in ALS. Further study of AMX0035 and TUDCA in the clinical trial is currently underway (Table 1).

**Table 1:** Current available major medications.

Drugs	Effect	Mechanism of action	Side reaction
Riluzole	Prolongs survival	Inhibition of glutamate release. Stabilization of neuronal membranes and decrease in neuronal excitability	Liver function enzymes, gastrointestinal symptoms, malaise or weakness, decreased lung function, neutropenia.
Edaravone	Inhibit motor deterioration	Protect neurons from oxidative stress damage.	Allergic reactions, bruises or contusions, gait disturbance, headache, and Dermatitis, decreased numbers of platelets, red blood cells, and white blood cells, and elevated creatine kinase levels.
PB-TURSO	Influencing ALS disease progression	Altered transcription, reduced neuroinflammation and improved cellular energy metabolism.	Abdominal pain, diarrhea, nausea, upper respiratory infection.
Qalsody	Neuroprotection	Antisense oligonucleotide against SOD1.	Pain, fatigue, arthralgia, increased leukocytes in cerebrospinal fluid

### 2.2 Combination applications of medicine

One study identified the combination of nebivolol and donepezil (nebivolol-donepezil) for the treatment of ALS by analyzing data based on the genetic information from ALS patients and the pharmacogenomic data from the drug. The results showed that nebivolol-donepezil significantly reduced cytokine levels in microglial cell lines, inhibited nuclear factor-κB nuclear translocation in HeLa cells, and significantly protected against the excitotoxicity-induced neuronal loss by modulating the PI3K-Akt pathway, and facilitated the differentiation of neural precursor cells to motor neurons (28). Some studies have synthesized Edaravone derivatives coupled with 1-aminoadamantane with alkylidene or hydroxypropyl groups and investigated their biological activity, and the compounds have been found to inhibit the lipid peroxidation and the calcium-associated mitochondrial permeability, block fast sodium currents in the neurons of central nervous system, and reduce the aggregation of FUS-protein in the typical mutant form of ALS, and have the potential to be optimized for using in the treatment of ALS (29). PXT864, a low-dose combination of aminocaproate and baclofen, can show the protection of neuromuscular junction and motor neuron integrity in the glutamatergic-injured primary neuron-muscle, suggesting that it may be a promising therapeutic strategy for ALS (30). These results suggest that combination applications of medicine may help to stop the progression of ALS disease, improve the quality of life and life expectancy of patients, and are very promising treatments.

## 3 Advances in emerging drug therapies: genetically targeted therapies

TDP-43 is an RNA-binding protein, and cytoplasmic TDP-43 aggregates are present in sporadic and familial ALS, in addition to familial ALS caused by mutations in *SOD1*, and its aberrant aggregation and aberrant function have been implicated in the pathogenesis of ALS (31, 32). Several antibodies have been developed to recognize the misfolded or exposed regions of *TDP-43*, especially in the RNA recognition motif domains. Some of these antibodies can induce the degradation of pathological TDP-43 by proteasome or autophagy, or reduce inflammation and improve the cognitive and motor function in animal models (33). Peptides derived from the C-terminal domain of TDP-43 have been designed to reduce TDP-43 aggregation by interfering with self-interaction or promoting clearance (34). Some of these peptides are conjugated with cell-penetrating or hydrophobic motifs to enhance their delivery and efficacy. Small molecules have been discovered or screened to bind to the different domains of TDP-43, such as the N-terminal domain, the RNA recognition motifs, or the C-terminal domain (33, 35). These molecules can modulate TDP-43 aggregation, degradation, or nucleic acid binding, and have shown the beneficial effects in cell and fly models of TDP-43 proteinopathy.

Antisense oligonucleotides (ASOs) are the synthetic nucleic acid molecules that can complementarily pair with a target mRNA to activate the endogenous RNase H enzyme, leading to the degradation of mRNA, or to block the interaction of mRNA with RNA-binding proteins, which regulates the shearing or processing of mRNA without degrading mRNA (36). MiRNA is a naturally occurring small molecule RNA that can partially complementarily pair with the 3′ untranslated region of the target mRNA to inhibit the translation of mRNA or promote the degradation of mRNA (36). Both approaches can be used to reduce or inhibit the expression of mutant genes, thereby reducing the production of mutant proteins, which may be an effective therapeutic strategy for some mutations with the toxic gain or loss of function. Both approaches have been used to target the *SOD1* and *C9orf72* genes in ALS therapy. MiRNA therapy targeting the *SOD1* and *C9orf72* genes has so far only shown some effects in animal models and *in vitro* cellular models (37–39). The ASO therapy targeting the *C9orf72* gene has completed a phase I clinical trial, but has not shown significant clinical benefit yet (40). The ASO therapy targeting the *SOD1* gene has entered phase III clinical trials but has not shown significant clinical benefit yet.

The RNA interference (RNAi) is a method of silencing specific genes using double-stranded RNA that can be used to reduce the expression of mutant proteins by fully or partially complementary pairing with the target mRNA, which can lead to the degradation or translational repression of mRNA (36). The RNAi molecules usually include the short interfering RNAs (siRNAs), the short hairpin RNAs (shRNAs) and the artificial miRNAs, which need to be delivered to the cell via the viral vectors (e.g., adeno-associated viruses) to function as gene silencers (41). In ALS, this approach has been used to the target mutations in the *SOD1* and *C9orf72* genes, both of which can lead to the degenerative changes and death of neurons (42, 43). The RNAi therapy targeting the *SOD1* gene has shown some effects in animal models, reducing the SOD1 protein levels and delaying the disease onset and progression (44–46). In humans, a study using the AAV9 vector to deliver an artificial miRNA targeting the *SOD1* gene in two patients with familial ALS found that it could safely inhibit *SOD1* transcription, reduce polypeptide dipeptide levels, and maintain the functional stability for 18 months (47). The RNAi therapy targeting the *C9orf72* gene has not entered clinical trials yet, but has shown some effects in the *in vitro* cellular and animal models, reducing *C9orf72* mRNA levels, RNA aggregation and polypeptide repeat protein production, and improving the neuronal survival and function (37–39).

The CRISPR/Cas is a technology that uses a repetitive sequence and associated proteins found in bacteria to enable gene editing, which can be done by designing specific single-stranded RNAs (sgRNAs) to direct the Cas enzyme to cleave the target DNA sequence, thereby repairing or knocking out the mutated DNA (48). In a study of CRISPR/Cas therapy targeting the *SOD1* gene using the AAV9 vector to inject SaCas9 and sgRNA targeting the *SOD1* gene into neonatal transgenic G93A-*SOD1* mice through a facial vein, it was found that it could significantly reduce the level of the mutant SOD1 protein, increase the number of motor neurons, delay the onset and progression of the disease, and improve survival rates (49). The CRISPR/Cas therapy targeting the *C9orf72* gene was found to reduce the transcription or levels of repetitive RNA, RNA aggregation and peptide repeat protein production in two studies targeting G4C2 repeat-expanded DNA or RNA, respectively (50, 51). This approach has the advantage of directly correcting the mutated DNA, thereby eliminating the downstream aberrant pathway, and could theoretically be used as a one-time intervention. However, the safety and feasibility of this approach has not been fully validated in humans, and a number of potential issues need to be addressed, such as the precision of gene editing, nonspecific side effects, effective vectors and delivery systems, and ethical and legal regulations.

Methods such as small molecule compounds or antibodies can also be used to intervene in the transcription process or to reduce the levels of mutant proteins, thereby attenuating or delaying the progression of neurodegenerative diseases, but there are more challenges involved. Some studies using methods such as small molecule compounds or antibodies are listed next, including: Interference with the secondary structure of repetitive RNA using compounds such as TMPyP4 (5,10,15,20-tetra (N-methyl-4-pyridyl) porphyrin), which reduces the RNA aggregation and peptide repeat protein production (52). The anti-GA antibody was used to inhibit the intra- and extracellular dissemination of GA peptides, thereby reducing cytotoxicity (53). Small molecule heat shock proteins such as heat shock protein B8 were used to promote the autophagic clearance of all five polypeptide repeat proteins, thereby improving the neuronal survival and function (54–56).

Approximately 2% of patients with ALS have mutations in the gene that encodes *SOD1*, a metalloprotease that protects against oxidative stress. These mutations lead to the dysregulation and overproduction of SOD1 protein (3, 57, 58). Under development by Biogen, Tofersen, known commercially as Qalsody™, is a therapeutic antisense oligonucleotide designed to target *SOD1* mRNA, with the goal of treating ALS. By targeting the *SOD1* mRNA, Tofersen works through an antisense mechanism. This mechanism involves binding to the mRNA sequence and triggering a process known as RNase H-mediated degradation. This degradation prevents the translation of *SOD1* mRNA into SOD1 protein, thereby lowering the amount of mutant SOD1 protein in affected cells (59). Tofersen was recently approved by the European Medicines Agency (EMA) and US FDA to use in treating SOD1 patients in April 2023. However, although Qalsody (Tofersen) performed well in terms of safety and tolerability and showed some degree of neuroprotection, it failed to meet the primary endpoints of reducing SOD1 protein levels and improving ALS functional scores in a phase III clinical trial (40).

## 4 Neuroprotective therapies

Antioxidants are a class of substances that resist oxidative stress and protect cells from damage caused by free radicals and other reactive oxygen species. Antioxidants may play an important role in the treatment of neurodegenerative diseases such as ALS, as oxidative stress is considered to be one of the main factors leading to neuronal death (60). There are many types of antioxidants, including endogenous (e.g., glutathione, superoxide dismutase, catalase, etc.) and exogenous (e.g., vitamin C, vitamin E, beta-carotene, polyphenols, etc.) (61). The efficacy of antioxidants has not been clearly demonstrated in clinical trials in ALS yet, but some antioxidants such as vitamin E, coenzyme Q10, melatonin and N-acetylcysteine have been used as adjunctive therapies in ALS or are undergoing further study (62–64). The safety and dosage of antioxidants are also factors to be considered, as excess antioxidants may have negative effects, such as inhibiting endogenous antioxidant defense systems, increasing oxidative stress, and interfering with the action of other drugs.

Mitochondria are the energy factories of cell and are involved in a variety of life activities such as the respiratory chain, calcium homeostasis, reactive oxygen species (ROS) production and apoptosis. The mitochondrial dysfunction leads to the motor neuron degeneration and death. To protect mitochondria from oxidative damage, several mitochondria-targeted antioxidants have been developed, such as MitoQ. These compounds are able to selectively accumulate in mitochondria, scavenge ROS, improve mitochondrial function, and delay the course of disease in animal models of ALS (65). The mitochondrial quantity and quality are regulated by the mitochondrial biosynthesis and clearance, including the mitochondrial dynamics and the mitochondrial autophagy. In ALS, all these processes are affected, leading to the disorganization of the mitochondrial network and accumulation of damaged mitochondria. Some compounds that modulate the mitochondrial biosynthesis and clearance, such as Olesoxime and Triheptanoin, have been evaluated as the potential therapeutic agents for ALS, but the results of clinical trials have been unsatisfactory (66, 67).

## 5 Stem cell therapies

**Figure 1:**
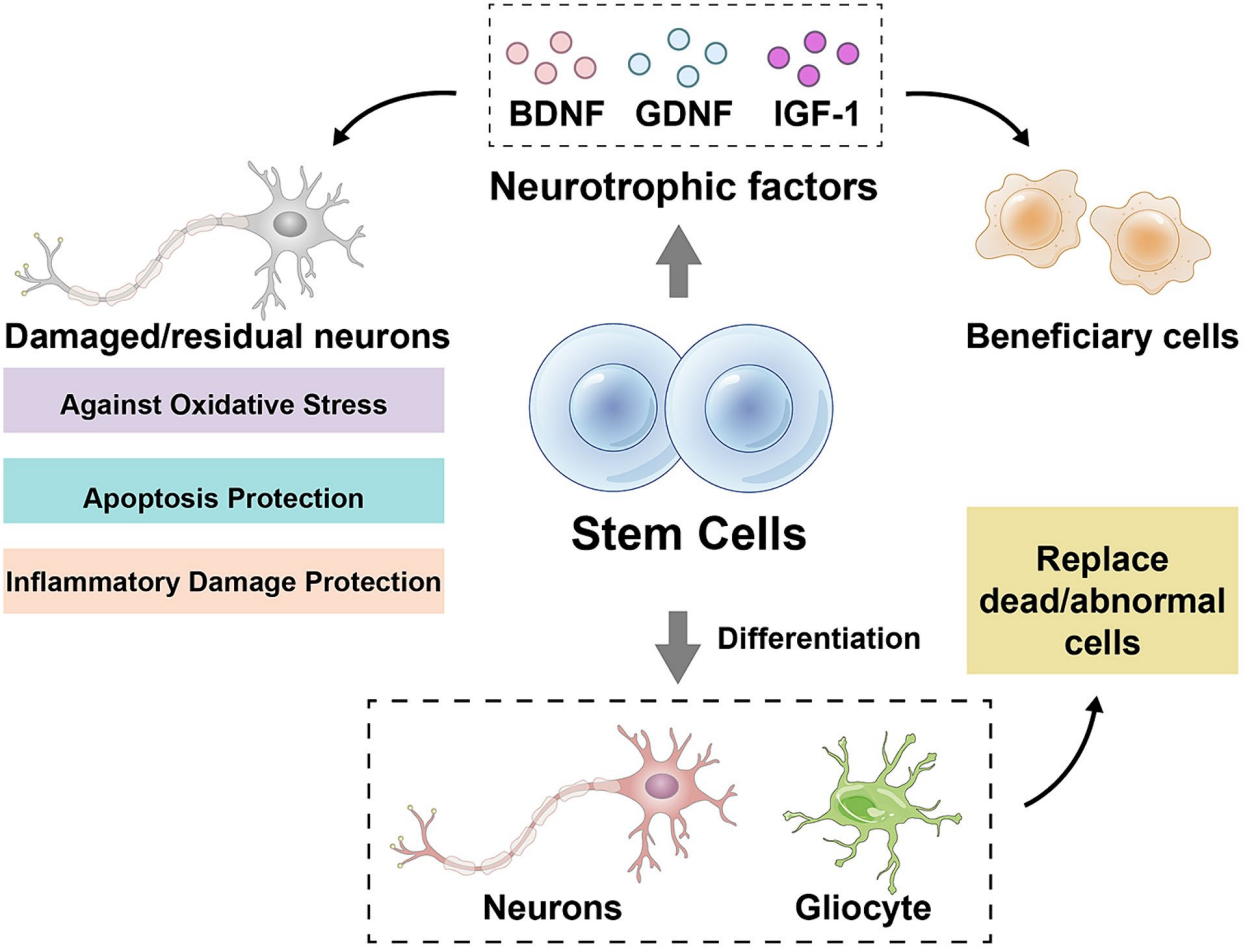
Mechanisms of stem cell action. BDNF, brain-derived neurotrophic factor; GDNF, glial cell-derived neurotrophic factor; IGF-1, insulin-like growth factor-1. Drawn by Figdraw.

The stem cell therapy is a method using stem cells or their derivatives to repair or replace the damaged nerve tissue with a view to the slowing or reversing progression of ALS. Currently, two main types of stem cells are used for ALS treatment, neural stem cells (NSCs) and mesenchymal stem cells (MSCs). NSCs are the pluripotent cells being capable of differentiating into neurons, astrocytes and oligodendrocytes, whereas MSCs are the pluripotent cells being capable of differentiating into a wide range of mesenchymal tissues, such as bone, cartilage, muscle and fat. Stem cells can protect the damaged or residual neurons against oxidative stress, apoptosis and the inflammatory damage by secreting neurotrophic factors such as brain-derived neurotrophic factor, glial cell-derived neurotrophic factor and insulin-like growth factor-1 (68–72) (Figure 1). Stem cells can replace dead or dysfunctional cells by differentiating into neurons or glial cells, restoring the structure and function of neural networks (69, 70, 73, 74) (Figure 1). Stem cells can also inhibit the onset and progression of neuroinflammation and attenuate the immune-mediated injury in neurodegenerative diseases by influencing the activation, differentiation, migration and secretion of immune cells (69, 70, 73, 74). Several clinical trials are currently underway or have been completed to evaluate the safety and efficacy of NSCs or MSCs in the patients with ALS. The transplantation of NSCs is usually done by the surgical injection of cells into spinal cord or brain ventricles, whereas the transplantation of MSCs can be done by intravenous or intraspinal injections. The source of NSCs and MSCs can be embryonic stem cells, adult stem cells, or induced pluripotent stem cells, and can be autologous or allogeneic.

There are a number of stem cell treatments that have entered clinical trials. A study included 20 subjects with a confirmed diagnosis of ALS and ALSFRS-R score of >20, who received intrathecal MSC injections 1–4 times at 3–6 month intervals, with the primary endpoints being safety and tolerability, efficacy assessments included ALSFRS-R score and forceful lung capacity (FVC) as secondary endpoints, results in no serious adverse events observed throughout the trial (75). A study evaluated the safety and efficacy of autologous bone marrow-derived mesenchymal stem cells (BM-D MSCs) (76), the results showed that treatment with BM-D MSCs slowed the disease progression in ALS patients with an inherently rapid disease course, However, due to the small size of this group, it wasn’t possible to assess whether these changes were statistically significant. A phase I clinical trial that included 18 patients with ALS validated the feasibility and safety of stem cell transplantation by microinjecting human neural stem cells (hNSCs) into the patients’ lumbar spine or cervical medullary gray matter bundles to evaluate hNSCs and monitoring the patients’ clinical, psychological, neuroradiological and neurophysiological profiles before and after transplantation (77). None of these patients exhibited serious adverse effects or accelerated disease progression as a result of the treatment for up to 60 months postoperatively, which suggests that the transplantation of hNSCs did not cause any long-term complications and that some patients showed the temporary clinical improvement after transplantation.

Although stem cell therapy has shown some safety and efficacy in clinical trials for ALS, a number of challenges and limitations remain. There are numerous factors that influence the effectiveness of stem cell therapy and there are no uniform standards or guidelines to regulate these factors. The transplantation of stem cells can be risky, with difficulties such as the financial burden, cell supply and heterogeneity.

## 6 Supportive treatment

**Figure 2:**
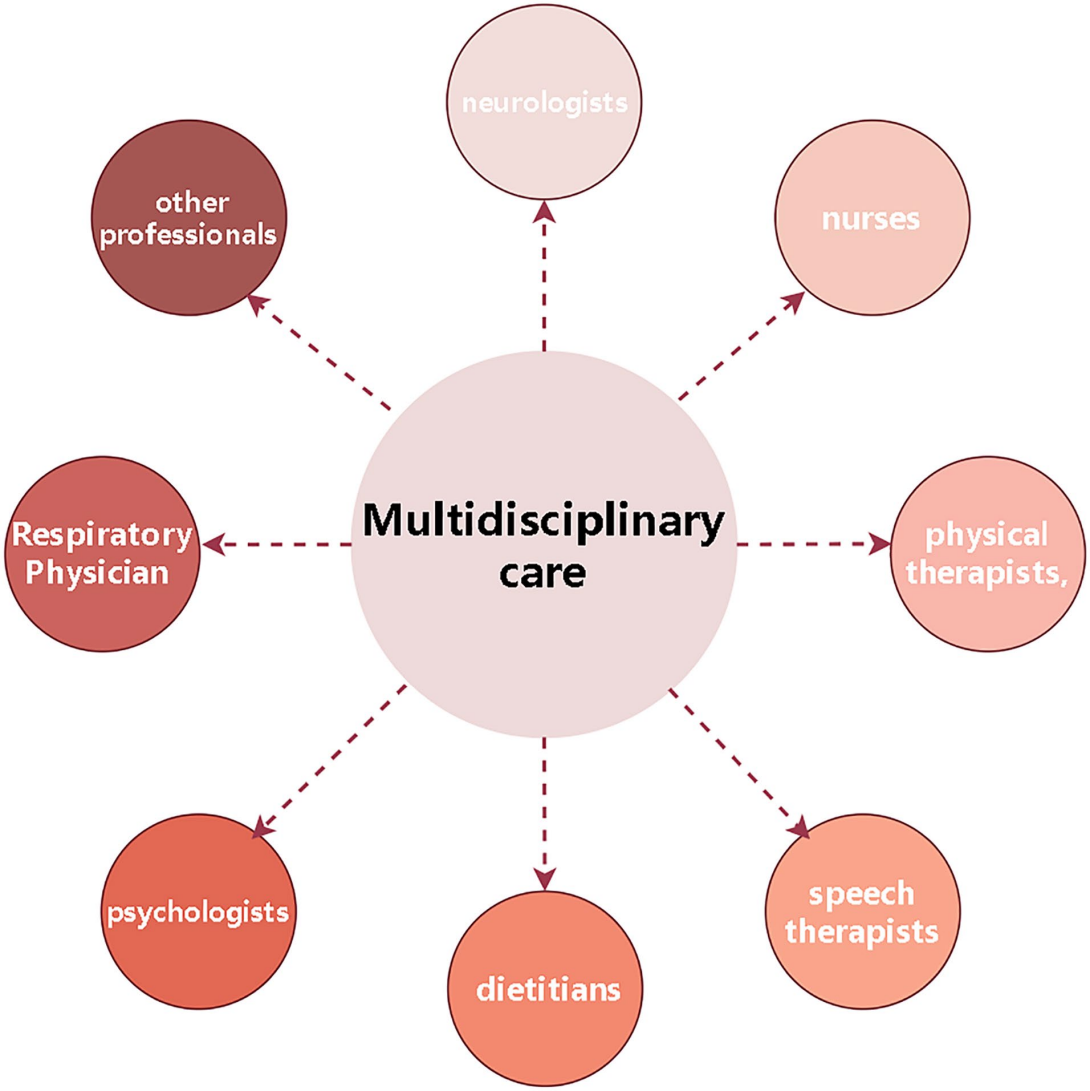
Multidisciplinary care. Drawn by Figdraw.

In addition to medications, the supportive treatment is an important component of treatment for ALS patients, including physical, occupational and speech therapy, nutritional and respiratory therapy, and psychological and palliative treatment. The supportive treatment not only relieves symptoms caused by ALS, but also improves quality of life and functional ability. The multidisciplinary treatment is the foundation of ALS treatment, involving neurologists, nurses, physical therapists, speech therapists, dietitians, psychologists and other professionals to provide a comprehensive medical and psychological support for ALS patients to improve quality of life and prolong survival (78–80) (Figure 2).

### 6.1 Non-motor symptoms treatment/symptomatic treatments for ALS

In the absence of cure or any medical intervention which might reverse or stop the progression of ALS, Therefore, the focus on the symptom, rehabilitation and palliation treatment with overall aim of optimizing quality of life is especially important. Among them, the treatments that can release/alleviate symptoms but do not target the pathogenic processes of ALS are included those that target ALS at the impairment level, such as the mechanical ventilation for respiratory insufficiency, the enteral feeding for maintaining the nutrition impairment, and the treatments of spasticity, sialorrhoea (autonomic nerves impairment), cramps and pain (sensory pain), and those that target ALS at the level of activity and participation, such as multidisciplinary care, repetitive transcranial magnetic stimulation (rTMS) and therapeutic exercise. A wide range of interventions is used in treating the diverse symptoms and impairments including cognitive-behavioral treatment in ALS.

#### 6.1.1 At the level of impairment, interventions include the following

1. The mechanical ventilation of tracheostomy and non-invasive ventilation might prolong the survival and optimize quality of life by ventilation in those with clinically significant respiratory muscle weakness (81).
2. The enteral feeding might maintain the bodyweight balance, prolong the survival, and improve the quality of life by providing a safe and reliable route nutrition in ALS patients who may have dysphagia, poor appetite and impaired ability to feed themselves to lead to the reduction of oral intake, malnutrition and dehydration.
3. The spasticity treatment is very widely, including the physiotherapy such as therapeutic exercises, stretching and positioning; The modalities such as heat, cold, vibration and electrical stimulation; The prescription medication such as baclofen; The non-prescription medication such as vitamins; The chemical neurolysis such as botulinum toxin; The surgical interventions such as intrathecal pumps and the alternative therapies such as reflexology. How these interventions might work varies widely from one intervention to another, most commonly, stretching techniques are used in combining with one or more ‘true’ muscle relaxants line baclofen (82), and such interventions work by lengthening with or without the assistance of weakening the muscle agonist.
4. The sialorrhoea treatments include suction, drug treatments and more invasive approaches, such as injecting botulinum toxin or irradiating salivary glands, which may improve sialorrhoea secretion and quality of life. These intervention measures reducing the amount of saliva either through its removal such as suction or reducing salivary secretion such as anticholinergic medication and botulinum toxin injection. The autonomic symptoms occur in majority of ALS patients to late stages over time, which implies that the autonomic dysfunction represents an intrinsic non-motor feature of ALS. A higher autonomic burden is a poor prognostic risk factor, being associated with a more rapid development of disease and shorter survival (83).
5. The cramps treatment, because the pathogenesis of cramps is not very clearly known yet, the mechanisms of treatment aren’t clear yet. Currently, 2 different pathophysiological mechanisms have been proposed, the abnormal excitation of terminal branches of motor axons (84), and the hyperexcitability or bistability of motor neurons in spinal cord (85). Therefore, the etiology of cramps in ALS and the mechanism of treatment measures remains uncertain yet.
6. The pharmacological treatment of pain in ALS, the present treatment for pain mainly reduces the pain symptom. Analgesics exert via different pathways. Such as paracetamol and non-steroidal anti-inflammatory drugs inhibit the production of pain by inhibiting the production of prostaglandins, while opiates such as morphine imitate natural neuromediators by binding their receptors such as endorphin receptors to relieve pain.
7. The cognitive-behavioral treatment. Approximate 50% of ALS patients experience the mild cognitive-behavioral changes. The cognitive impairment can lead to the increased variability in gait parameters and a higher risk of falls (86). The behavioral disturbances may result in the refusal of necessary therapeutic interventions in the advanced stages of ALS, such as tracheostomy or PEG tube placement (87). ALS patients with executive dysfunction have a worse prognosis, and the behavioral changes have a negative impact on the carer quality of life (88, 89). Screening for cognitive deficits is recommended, because it can provide the patients and carer support (90–92). Standard memory tests, such as Mini-Mental State Examination, ALS-Cognitive Behavioral Screen and more extensive University of California San Francisco Screening Battery (93), and the Edinburgh Cognitive Assessment Screen (94) are recommended in clinical cognitive estimation. Moreover, other types of neurological dysfunction including ataxia and autonomic dysfunction also should be estimated. Currently, no accurate evidences exist to guide the management of cognitive or other neurological deficits in ALS, and establishing the evidence-based strategies to manage such ALS cognitive-behavioral symptoms should be a priority for further research.

Approximate half of ALS patients experience psychological symptoms, such as emotional lability, pathological laughter or crying, these symptoms are more common in the ALS patients with bulbar onset ALS (95). The compounds of dextromethorphan combining with quinidine can improve emotional symptoms (96, 97). In addition, selective serotonin reuptake inhibitors such as citalopram and amitriptyline (91, 98, 99) also can be used in treating emotional symptoms such as depression and anxiety. The psychological symptoms are common including depression, anxiety and fatigue, and have a negative effect on quality of life (100–102). Modafinil can be safely used for fatigue (103). Otherwise, psychological support, palliative care and physical therapy is recommended (92), along with standard drug treatments used in other diseases (104).

#### 6.1.2 At the level of activity and participation, interventions include the following

1. Multidisciplinary care might reduce disability and improve quality of life by applying “a problem-solving education process” (105), delivered by medical and allied health disciplines, for example, physiotherapy, occupational therapy and speech therapy, which are focused on maximizing activity and participation.
2. Transcranial magnetic stimulation might stimulate nerve cells in superficial areas of brain by applying a high-energy magnetic field at the skull surface which induces a perpendicular electrical field in the vertical plane through cerebral cortex, which might provide a non-invasive approach to condition the excitability and activity of neurons (106), and at the low frequency of equal to or less than 1 Hz, bring a reduction in glutamate-induced excitotoxicity, which may improve motor function in ALS patients (107). At the higher frequency of faster than 1 Hz, it is thought that the increased expression of neurotrophic factors could be neuroprotective (108). In the last 20 years, several modalities of neuromodulation, mainly based on non-invasive brain stimulation (NIBS) techniques, have been tested as a non-pharmacological therapeutic approach to slow disease progression in ALS. In both sporadic and familial ALS patients, the neurophysiological studies pointed to the motor cortical hyperexcitability as a possible priming factor in neurodegeneration, likely related to the dysfunction of both excitatory and inhibitory mechanisms. A trans-synaptic anterograde mechanism of excitotoxicity is thus postulated, causing upper and lower motor neuron degeneration. Specifically, both motor neuron hyperexcitability and hyperactivity are attributed to intrinsic cell abnormalities related to alter the ion homeostasis and to impair the glutamate and gamma aminobutyric acid gamma-aminobutyric acid signaling. Several neuropathological mechanisms supported both excitatory and synaptic dysfunction in ALS. In addition, corticospinal excitability can be suppressed or enhanced using NIBS techniques such as rTMS and transcranial direct current stimulation (tDCS), as well as invasive brain and spinal stimulation. Experimental evidence supports the hypothesis that the after-effects of NIBS are mediated by long-term potentiation−/long-term depression-like mechanisms modulating synaptic activity, with different biological and physiological mechanisms underlying the effects of tDCS and rTMS and possibly different rTMS protocols. Overall, these studies suggest a possible efficacy of neuromodulation in determining a slight reduction of ALS progression, related to the type, duration and frequency of treatment, but current evidence remains preliminary (109, 110).
3. The therapeutic exercise might reduce disability and fatigue, and improve quality of life by improving cardiovascular deconditioning and disuse weakness in ALS patients.

## 7 Conclusion

The current mainstay of treatment for ALS remains comprehensive care and symptom management, and a number of medications are available to slow disease progression. Gene therapy and combination therapy is also a promising area of research, but is still in the experimental stage. The treatment of ALS remains challenging and further research and clinical trials are needed to find more effective treatments. Establishing more accurate animal models, exploring more genetic variants and biomarkers, and developing more effective and safe treatments may help to develop strategies for ALS treatment in the future. Recent studies employing skin biopsies have pointed out that when ALS patients are categorized according to King’s stages, there is a gradual rise in intraepidermal nerve fiber and a progressive decline in Meissner corpuscles throughout the clinical stages, aligning with the progression and severity of ALS. This evidence could be valuable in trials as a surrogate outcome to monitor ALS progression and treatment response (111). Based on literature screening criteria, although the pathogenesis of ALS has not very clear, the excitatory toxicity and oxygen free radicals lesion participates in the pathogenesis of ALS is universally acknowledged, the drugs on which the focus was placed were chosen based on these two key damaged mechanisms in ALS, the extensive available and approved drugs for the treatment of ALS in the worldwide only Riluzole and Edaravone, which are the inhibitor of excitatory toxic of glutamate neurotransmitter and the scavenger of oxygen free radicals, respectively. In addition, there are numerous trials undertaken and in progress regarding ALS treatments including medications, neuroprotection agents, gene therapeutic measures, and stem cells, such as PB-TURSO, AMX0035, TUDCA, nebivolol-donepezil, Edaravone derivatives, PXT864, Tofersen, Olesoxime, Triheptanoin, NSCs, and MSCs, etc. Among them, some trials are failed, some results of clinical trials have been unsatisfactory, some trails are in progress. Although several drugs are used in clinical treatment of ALS, and numerous trials undertaken regarding ALS treatments, no effective measures could reverse or stop the progression of ALS up to now. In the present clinical practice implication, the combination of multiple treatment measures to improve the quality of life and prolong survivals of ALS patients, including medicine and supportive treatment, especially symptomatic and supportive treatment for the quality of life and survival of patients especially are important in the absence of effective drugs at present. In our clinical experiences, we applied multiple measures to treat the ALS patients based on the current reported potential pathogenesis of ALS and improve the quality of life and partially extend the survival of ALS patients, including Riluzole and Edaravone as well as neuron protective drugs used in clinically treating other diseases such as stroke, immunological inhibitors such as clinically treating autoimmunological diseases, antivirus drugs, neuronutritive drugs such as vitamin E and B, neural growth factors, etc. Meanwhile, we combine the multidisciplinary care and the Chinese traditional medicine. This treatment is known by our-self as “Cocktail treatment,” which currently obtain partial effects in the partial ALS patients. Of course, our “Cocktail treatment” need further experimental and clinical evidences to support, and search more effective combination.

## Author contributions

LL: Conceptualization, Writing – original draft, Writing – review & editing. YD: Conceptualization, Writing – original draft, Writing – review & editing. RX: Conceptualization, Funding acquisition, Writing – original draft, Writing – review & editing.
